# Reported baseline variables in transsphenoidal surgery for pituitary adenoma over a 30 year period: a systematic review

**DOI:** 10.1007/s11102-023-01357-w

**Published:** 2023-10-16

**Authors:** Hugo Layard Horsfall, Ryan T. S. Loh, Ashwin Venkatesh, Danyal Z. Khan, Alistair Lawrence, Ronie Jayapalan, Olympia Koulouri, Daniele Borsetto, Thomas Santarius, Mark Gurnell, Neil Dorward, Richard Mannion, Hani J. Marcus, Angelos G. Kolias

**Affiliations:** 1 Division of Neurosurgery, Department of Clinical Neurosciences, Addenbrooke’s Hospital and University of Cambridge, Cambridge, UK; 2 Department of Neurosurgery, The National Hospital for Neurology and Neurosurgery, London, UK; 3 Wellcome/EPSRC Centre for Interventional and Surgical Sciences, University College London, London, UK; 4 School of Clinical Medicine, University of Cambridge, Cambridge, UK; 5 Department of Neurosurgery, Royal London Hospital, London, UK; 6 Metabolic Research Laboratories, Institute of Metabolic Science, University of Cambridge and Cambridge NIHR Biomedical Research Centre, Addenbrooke’s Hospital, Cambridge, UK; 7 Department of Otolaryngology, Addenbrooke’s Hospital and University of Cambridge, Cambridge, UK

**Keywords:** Neurosurgery, Pituitary, Adenoma, Core outcome sets, Common data elements

## Abstract

**Purpose:**

Heterogeneous reporting in baseline variables in patients undergoing transsphenoidal resection of pituitary adenoma precludes meaningful meta-analysis. We therefore examined trends in reported baseline variables, and degree of heterogeneity of reported variables in 30 years of literature.

**Methods:**

A systematic review of PubMed and Embase was conducted on studies that reported outcomes for transsphenoidal surgery for pituitary adenoma 1990–2021. The protocol was registered a priori and adhered to the PRISMA statement. Full-text studies in English with > 10 patients (prospective), > 500 patients (retrospective), or randomised trials were included.

**Results:**

178 studies were included, comprising 427,659 patients: 52 retrospective (29%); 118 prospective (66%); 9 randomised controlled trials (5%). The majority of studies were published in the last 10 years (71%) and originated from North America (38%). Most studies described patient demographics, such as age (165 studies, 93%) and sex (164 studies, 92%). Ethnicity (24%) and co-morbidities (25%) were less frequently reported. Clinical baseline variables included endocrine (60%), ophthalmic (34%), nasal (7%), and cognitive (5%). Preoperative radiological variables were described in 132 studies (74%). MRI alone was the most utilised imaging modality (67%). Further specific radiological baseline variables included: tumour diameter (52 studies, 39%); tumour volume (28 studies, 21%); cavernous sinus invasion (53 studies, 40%); Wilson Hardy grade (25 studies, 19%); Knosp grade (36 studies, 27%).

**Conclusions:**

There is heterogeneity in the reporting of baseline variables in patients undergoing transsphenoidal surgery for pituitary adenoma. This review supports the need to develop a common data element to facilitate meaningful comparative research, trial design, and reduce research inefficiency.

**Supplementary Information:**

The online version contains supplementary material available at 10.1007/s11102-023-01357-w.

## Introduction

Pituitary adenomas are common, benign tumours [1–3]. Treatment options include medical management, surgery, or radiotherapy [4]. Surgery almost always utilises the transsphenoidal approach to resect the pituitary adenoma [5], either microscopically or endoscopically. Recent improvements in imaging techniques and devices such as the endoscope, coupled with next generation artificial intelligence to predict post-operative response to medical therapy, have advanced pituitary adenoma treatment, including surgical management [4, 6–11] However, despite these advances, there remain important, unanswered questions in relation to pre-operative assessment, intraoperative techniques, and post-operative management. To address these questions, high quality data is required from robustly designed studies that permit cross-comparison and meta-analysis. Heterogenous data remains an issue for the global scientific community, and contributes to significant research wastage, inefficiency, and escalating costs of biomedical research [12].

The present systematic review aimed to establish the trends and degree of heterogeneity in the reported baseline data elements of patients undergoing transsphenoidal surgical resection of a pituitary adenoma. We anticipate this will form the first step in the development of an international collaborative common data elements (CDE), with the potential to enhance specialist pituitary clinical services and facilitate research to address outstanding questions relating to the pre-operative, peri-operative and post-operative management of pituitary adenoma patients [13, 14].

## Methods

### Protocol and registration

The protocol for this systematic review was registered prospectively with OSH Registries (www.osf.io; doi:10.17605/osf.io/v9a6j). This review was conducted in accordance with the Preferred Reporting Items for Systematic Reviews and Meta-Analyses (PRISMA) Guidelines [15].

### Search

A search of Medline and Embase databases was performed inclusive of 1990–2021 to identify studies containing pituitary adenoma, an intervention and outcome. We searched all studies describing the transsphenoidal approach for pituitary adenoma (Supplementary 1).

### Eligibility criteria

Randomized controlled clinical trials, prospective cohort studies (> 10 patients), and retrospective studies (> 500 patients) reporting patients undergoing operative transsphenoidal intervention as the primary treatment strategy were identified, using a previously described approach. A constraint based, pragmatic decision for retrospective studies of > 500 patients was made to assess which baseline data elements were reported and the overall trend, rather than specific variables. It is acknowledged that smaller studies with more granular data may be excluded and therefore induces potential bias. Case reports, studies describing medical-only treatment therapies, systematic reviews and studies reporting cranial operative approaches were excluded. Only studies written in English were included.

### Study selection

Assessment for eligibility was performed independently in duplicate by three authors (HLH, AL, RJ) in a blinded manner. Any disagreement was resolved by discussion—overseen by the senior author (AK).

### Data extraction

Data was extracted from full-text articles by the authors (RL, AV and HLH) using a piloted proforma Microsoft Excel (Microsoft Inc., Seattle, WA). The first author (HLH) verified the extracted data for every 10th paper included to ensure internal validity. Baseline data for each study were collected and are listed below (“data items”).

### Data items

The following information was extracted from each included study: (1) study details: first author, year, journal, location of study; (2) study design: study period, type of study; (3) patient demographic data: age, sex, body mass index, ethnicity, previous pituitary adenoma surgery, co-morbidities; (4) tumour histopathology: non-functioning, functioning (growth hormone, ACTH, prolactin, TSH, pluripotent); (5) clinical presentation baseline variables: endocrine, ophthalmic, nasal, cognitive, headache (6) preoperative imaging: imaging modality, radiological characteristics; (7) other baseline variables: surgical technique, ENT collaboration, use of intraoperative adjuncts.

### Analysis

Descriptive statistical analysis was performed using Microsoft Excel (Microsoft Inc., Seattle, WA).

### Risk of bias assessment

No assessment of the methodological quality of the included studies was performed as there was no synthesis of results data.

## Results

### Study demographics

**Fig. 1:**
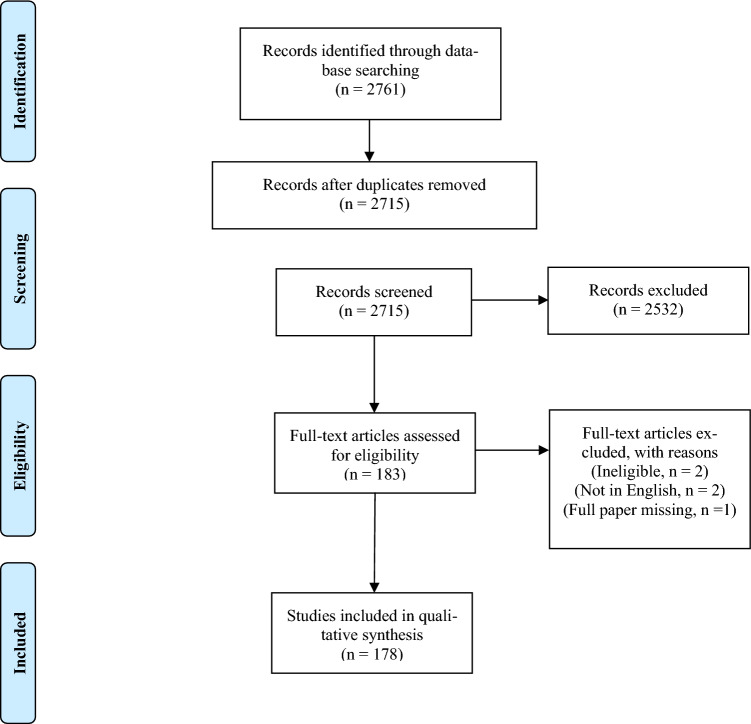
PRISMA Flow Diagram demonstrating inclusion of studies

A total of 178 studies were eligible for inclusion, comprising 427, 659 patients (Fig. 1). There were 52 retrospective studies (29%), 118 prospective studies (66%) and 9 randomised controlled trials (5%). One study included both retrospective and prospective patients. The number of studies reporting on transsphenoidal surgery over time has increased, from 14 studies in the years 1990–1999, to 36 studies in 2000–2009, and 129 studies in 2010–2021 (Table 1). North America was the continent with the most studies (67, 38%), whilst Europe and Asia had 50 and 48 studies respectively.

**Table 1:** Breakdown of studies which met the inclusion criteria by decade, number of patients and study type

Decade & study type	1990–1999			2000–2009			2010–2021			Total
No. of patients	R	P	RCT	R	P	RCT	R	P	RCT	
1–250	0	9	2	0	19	3	0	73	4	110
251–500	0	0	0	0	1	0	0	6	0	7
501–1000	2	0	0	5	2	0	18	6	0	33
> 1000	1	0	0	5	1	0	21	1	0	29
Total	3	9	2	10	23	3	39	86	4	179

R—retrospective cohort study, P—prospective cohort study, RCT—randomised controlled trial. Note a 2004 study with > 1000 patients included both retrospective and prospective data, and has been represented as two separate studies in this table. This table has been adapted from our previous work as identical inclusion criteria were used [16]

### Patient demographics

The majority of studies reported descriptive characteristics (age: 165 studies, 93%; sex: 164 studies, 92%) (Table 2). Forty-five studies (25%) reported patient co-morbidities and forty-two studies (24%) reported if the patient had undergone previous pituitary adenoma surgery, respectively. Only 21 studies (12%) reported BMI and 16 studies (9%) reported patient ethnicity (Table 3).

**Table 2:** Summary of data elements reported related to patient demographics, tumour histopathology and number of histopathological subtypes reported in the studies

Data element	Studies reporting data element	% of total studies (%)
Patient demographics		
Age	165	93
Sex	164	92
BMI	21	12
Ethnicity	16	9
Co-morbidities	45	25
Previous pituitary adenoma surgery	42	24
Tumour histopathology*		
Non-functioning	101	57
Functioning but does not specify	9	5
GH	106	60
ACTH	95	53
Prolactinoma	80	45
TSH	31	17
Pluripotent	6	3
Does not specify	27	15
Number of histopathological subtypes		
1	53	30
2	16	9
3	8	4
4	39	22
5	28	16

*BMI* body mass index, *GH* growth hormone, *ACTH* adrenocorticotrophin hormone, *TSH* thyroid stimulating hormone

*Please note that tumour histopathology states the number of studies that reported the specific pituitary pathology within the study, but that many studies reported more than one pathology within a study

**Table 3:** Baseline variable reporting (absolute number and % of total studies) across two different eras (1993–1998 & 2016–2021)

	1993–1998 (13 studies, 3187 patients)		2016–2021 (93 studies, 278272 patients)	
Baseline Variable	n	% n	n	% n
Study design	13	100	80	86
Patient demographics	12	92	82	88
Tumour pathology	11	85	82	88
Clinical presentation	9	69	47	51
Endocrine presentation	4	31	50	54
Pre-operative imaging	10	77	58	62
Peri-operative variables	10	77	81	87

### Tumour histopathology

The most commonly reported tumour histopathology was non-functioning (101 studies, 57%) and GH-secreting (106 studies, 60%) (Table 1). Thirty-six studies (20%) did not specify the tumour pathology. The studies that did not report a specific pathology often wrote ‘pituitary adenoma’, ‘functioning’ or ‘hormone secreting tumour’. Ninety-one studies (51%) reported 2 or more tumour histopathological subtypes in the series.

### Clinical baseline variables

**Fig. 2:**
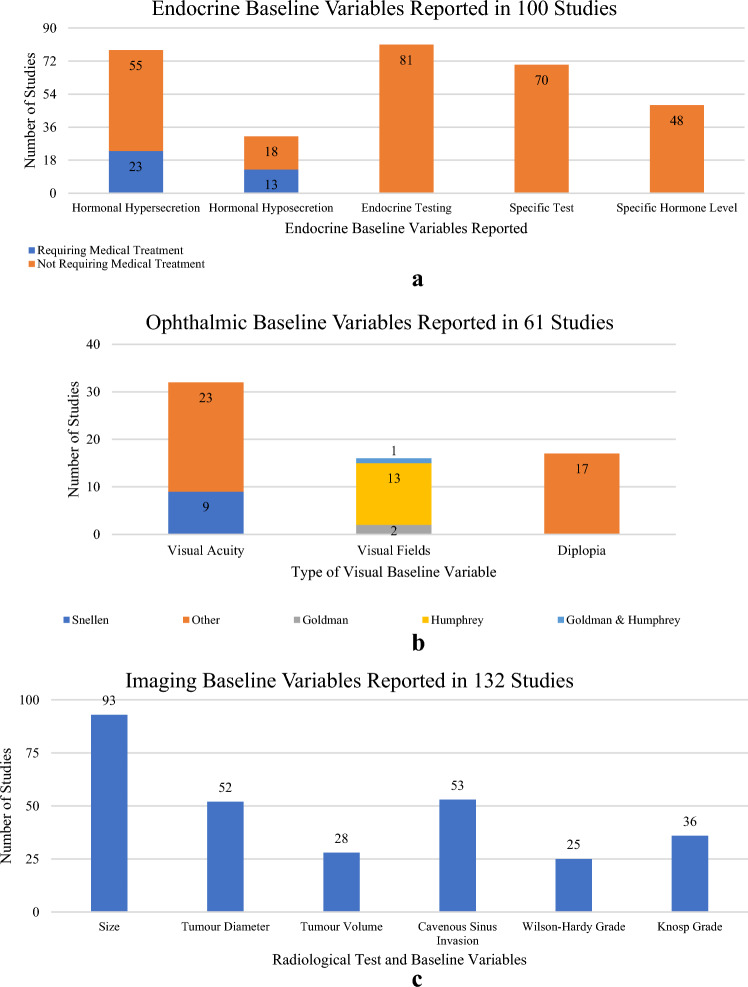
Summary of endocrine, ophthalmic, and radiological baseline variables. **a** Summary of endocrine baseline variables. **b** Summary of ophthalmic baseline variables. **c** Summary of radiological baseline variables

Pre-operative clinical presentation data included endocrine, ophthalmic, nasal, cognitive, or headaches (Fig. 2). Endocrine baseline variables were reported in 100 studies (56%; Fig. 2a). Evidence of hormonal hypersecretion was reported in 78 studies (44%) and hyposecretion in 31 studies (17%). Of the 101 papers reporting functioning adenomas, 37 studies (37%) reported hypersecretion and 29 studies (29%) reported hyposecretion. Of the 132 papers reporting non-functioning adenomas, 68 studies (52%) reported hypersecretion, while 22 studies (17%) reported hyposecretion. 88 studies included both functioning and non-functioning adenomas. 81 studies (46%) mentioned endocrine testing with 70 (39%) of them reporting or describing the specific test performed. 48 studies (27%) explicitly defined the hormone concentration. 13 studies (7.3%) reported patients who required pre-operative hormonal replacement due to one or more hormonal deficits. 23 (13%) studies reported patients who required medical suppression therapy pre-operatively.

For ophthalmic baseline variables, visual fields were reported in 61 studies (34%) and visual acuity in 32 studies (18%; Fig. 2b). Visual field was reported slightly more frequently in studies reporting functioning adenomas (42 studies, 42%), compared to studies reporting non-functioning adenomas (46 studies, 35%). Visual acuity was reported in 22 studies (22%) reporting functioning tumours, and in 24 studies (18%) reporting non-functioning tumours. For visual fields, the specific modality used was only reported in 16 of the 61 studies (26%): Humphrey (13 studies), Goldman (2 studies), both (1 study). Use of the Snellen Chart to assess acuity was reported in 9 of the 32 studies. Diplopia was reported in 17 studies (10%).

Nasal baseline variables were reported in 13 studies (7%) and a specific measure was reported in 10 studies: SNOT-22 (4 studies), Sniffin’ Sticks (3 studies), BAST-24 (1 study), (UPSIT, 1 study), NSS (1 study), and SIT40 (1 study). Cognitive baseline variables were reported in 9 studies (5%) and included mental disorders, altered mental status, depression, or mood change. One study that reported cognitive presentation used a validated measurement tool – the Mini Mental State Exam. Headaches were reported in 45 studies (25%).

### Imaging baseline variables

Preoperative imaging was reported in 132 studies (74%; Fig. 2c). MRI alone was the most reported imaging modality (89 studies, 67%), with 33 studies (25%) reporting both CT and MR imaging. CT alone was reported in 3 studies (2%). Only 12 studies (10%) detailed a specific pituitary imaging protocol. Designation of tumours as micro-/macro-adenomas was reported in 93 studies (70%). Of the studies that reported macroadenomas, 44 studies defined macroadenoma explicitly as > 10mm. Further specific radiological baseline variables reported included: tumour diameter (52 studies, 39%); tumour volume (28 studies, 21%); cavernous sinus invasion (53 studies, 40%); Wilson Hardy grade (25 studies, 19%); Knosp grade (36 studies, 27%; Fig. 2c). Eight studies (4%) reported the surgeon’s preoperative resection intention.

### Other baseline variables reported

The transsphenoidal approach was used in all studies. The endoscopic transsphenoidal technique was used in 78 studies, microscopic technique in 58 studies but 58 studies did not report the specific technique used to resect the pituitary adenoma. 30 studies reported collaboration with Ear, Nose and Throat surgeons. 37 studies (21%) reported the use of pre/intra- operative adjuncts. Of these, 13 studies reported insertion of a pre-operative lumbar drain. Other intra-operative adjuncts utilised were intraoperative CT (3 studies); intraoperative MRI (14 studies); intraoperative Doppler (2 studies); and neuronavigation (13 studies).

### Heterogeneity in baseline variables across two eras (1993–1998 & 2016–2021)

Comparing 1993–1998 with 2016–2021, study design was reported in 100% vs 86% respectively, patient demographics 92% vs 88% respectively, tumour pathology 85% vs 88% respectively, clinical presentation 69% vs 51% respectively, endocrine baseline variables 31% vs 54% respectively, pre-operative imaging 77% vs 62% respectively, and peri-operative variables 77% vs 87% respectively. These first and last five-year epochs were chosen to assess if there had been a change in reporting over time.

## Discussion

### Principal findings

This systematic review of 178 studies (comprising 427,659 patients over a 30-year period) has identified significant heterogeneity in reporting trends for baseline variables in patients with a pituitary adenoma undergoing transsphenoidal surgery. Even well-established baseline patient demographics such as BMI or co-morbidities were poorly reported (just 24% and 25% of studies respectively). Similarly, endocrine baseline variables were only reported in 60% of studies and ophthalmic baseline variables in 34% of studies, despite endocrine and ophthalmic presentations being important in the assessment of a pituitary adenoma. Pituitary adenoma management is complex and requires specialist multi-disciplinary input from the time of initial patient presentation through long-term follow up. The issue of heterogeneity in pituitary adenoma research is compounded due to multiple tumour types and biochemical characteristics. Similarly, not all patients with pituitary adenoma undergo transsphenoidal surgery. It may therefore be beneficial to consider a CDE in the context of each specific pathological subtype (and even the selected treatment arm) to permit the most important baseline variables to be considered.

### Findings within the context of the literature

There has been an increasing trend to standardise data collection and reporting requirements to facilitate data discovery, data interpretation, and data reuse [17]. CDEs provide structured, standardised definitions so that data may be collected and used across different datasets. CDE collections are traditionally developed prospectively by subject-matter and domain experts and are commonly used to define case report forms (CRFs) for clinical trials [17]. CDEs can also be used in any situation where it is important to meet rigorous data collection or reporting requirements [18]. The National Institute of Neurological Disorders and Stroke (NINDS) created the CDE project in 2006, to develop standards for performing funded neuroscience-related clinical research such as epilepsy, traumatic brain injury, and stroke [19–21]. These endeavours promise improved data management, accelerated research, and empower academics in resource-poor settings [10] to produce high-quality research that is internationally comparable due to homogenous data collection. This standardisation would benefit the management of pituitary adenoma, and previous work undertaken by our group has investigated the heterogeneity in outcomes [16]. Despite the exponential increase in research, there remain common issues that are poorly quantified. These high-quality studies require a common language, such as CDE, to help facilitate study design. Similarly, standardised endocrine baseline variable reporting would be beneficial.

### Limitations

This study has several limitations. First, due to heterogenous reporting and grouping together of numerous pituitary pathologies, the difficulty to ascertain more granular data on the baseline variables in transsphenoidal pituitary adenoma surgery. This was mitigated by multidisciplinary (neurosurgeons, endocrinologists, ear nose and throat surgeons) discussion about key baseline variables to pragmatically overview baseline variable reporting trends and heterogeneity over the last 30 years. The 30 year study period could introduce bias as diagnostic and therapeutic tools would have changed over time, and with it different measurement items and treatment strategies. This does risk the introduction of reporting bias. Additionally, we also provided the breakdown comparison from the first and last six years of the 30-year period to attempt to establish reporting trends over time. Indeed, depending on whom the senior author was of each study, their specialty (i.e. endocrinologist, neurosurgeon, ophthalmologist) may affect the focus of each study and reported baseline variables, which again supports homogenous data reporting would be hugely beneficial, achieved through a consensus-derived CDE with multiple specialties’ input.

## Conclusion

This systematic review of transsphenoidal pituitary adenoma surgery demonstrates heterogeneity in reporting of baseline variables over the last 30 years. In identifying heterogeneity, this study is a call to action for further work to develop an international, collaborative, consensus driven, common data element set for the transsphenoidal surgical management of pituitary adenoma. This would likely enhance data collection, improve comparative research, reduce research inefficiency and lead to improved patient outcomes.

### Supplementary Information

Below is the link to the electronic supplementary material.

Supplementary file1 (DOCX 16 kb)

## Data Availability

The datasets generated during and/or analysed during the current study are available from the corresponding author on reasonable request.

## Abbreviations

ACTH: Adrenocorticotrophic hormone
BMI: Body mass index
CDE: Common Data Element
CRF: Case report form
CSF: Cerebrospinal fluid
CT: Computed tomography
ENT: Ear, nose, throat
GH: Growth hormone
MRI: Magnetic resonance imaging
NINDS: National Institute of Neurological Disorders and Stroke
PRISMA: Preferred Reporting Items for Systematic Reviews and Meta-Analyses
TSH: Thyroid-stimulating hormone
